# Leap and strike kinetics of an acoustically ‘hunting’ barn owl (*Tyto alba*)

**DOI:** 10.1242/jeb.107169

**Published:** 2014-09-01

**Authors:** James R. Usherwood, Emily L. Sparkes, Renate Weller

**Affiliations:** The Structure and Motion Laboratory, The Royal Veterinary College, North Mymms, Hatfield, Hertfordshire AL9 7TA, UK

**Keywords:** Hunt, Jump, Owl, Power, Work

## Abstract

Barn owls are effective hunters of small rodents. One hunting technique is a leap from the ground followed by a brief flight and a plummeting ‘strike’ onto an acoustically targeted – and potentially entirely hidden – prey. We used forceplate measurements to derive kinetics of the leap and strike. Leaping performance was similar to reported values for guinea fowl. This is likely achieved despite the owl's considerably smaller size because of its relatively long legs and use of wing upstroke. Strikes appear deliberately forceful: impulses could have been spread over larger periods during greater deflections of the centre of mass, as observed in leaping and an alighting landing measurement. The strike, despite forces around 150 times that of a mouse body weight, is not thought to be crucial to the kill; rather, forceful strikes may function primarily to enable rapid penetration of leaf litter or snow cover, allowing grasping of hidden prey.

## INTRODUCTION

**Fig. 1.**
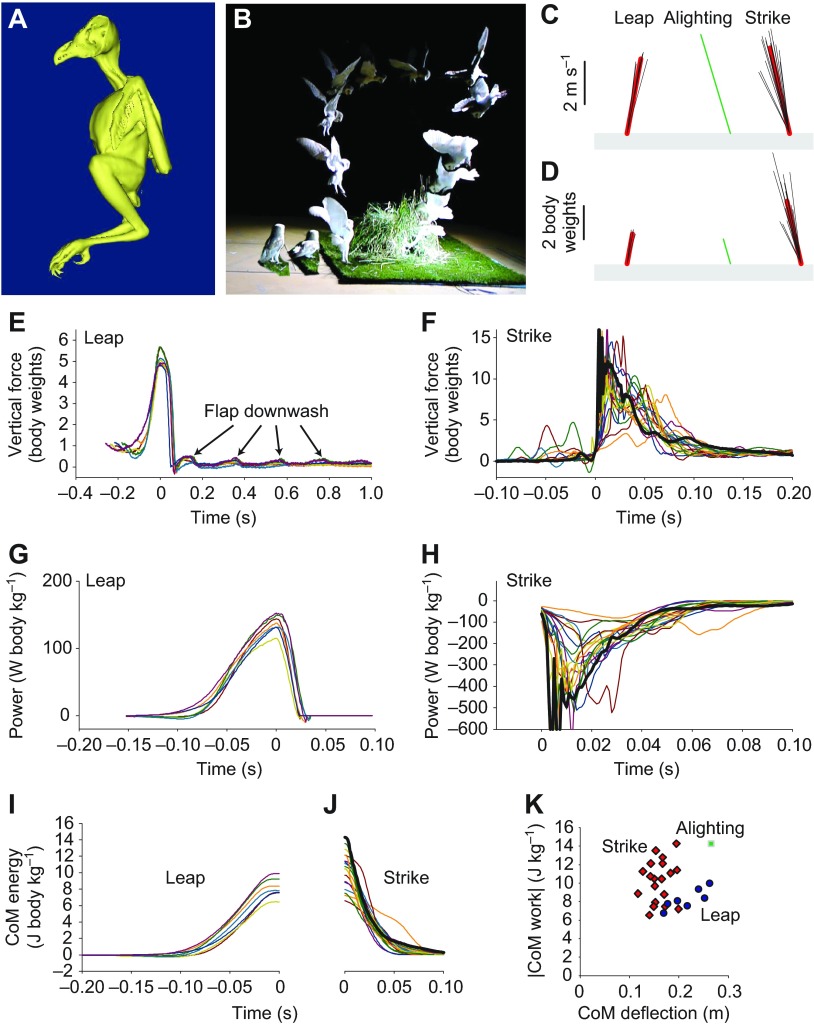
**Barn owl morphology and hunting kinetics.** (A) Lateral view of a 3D model of a barn owl based on computed tomographic images illustrating the relatively, though not excessively, long leg length of this species. (B) Forceplate-derived kinetics of a barn owl leaping (*N*=7), alighting (*N*=1) and pouncing onto or ‘striking’ a stiff plastic beeper box hidden within a ring of long grass, mounted to a forceplate top plate (*N*=19). Impulses (shown in mass-specific form, C) and mean force vectors (D) during active leg pushing phases (see Materials and methods for timing definitions) were predominantly vertical. Vertical forces (E,F), centre of mass (CoM) powers (G,H) and changes in energy (I,J) through time are shown for periods close to leap (E,G,I) and strike (F,H,J). Note the different scales used in E–H. The bold black line (F,H,I) denotes the most energetic strike trial. CoM vertical deflection and work during active leg pushing (K) show that the owl effectively falls further than it jumps, and, while it has the ability to flex its legs to compensate during landing, it does not. Thus, considerably higher forces and powers are produced during the more energetic strikes compared with leaping or alighting.

Barn owls, *Tyto alba* (Scopoli 1769), are distinctively long-legged birds (Fig. 1A) (Cramp, 1985), and are capable hunters, predominantly of mice, voles and shrews [summarized in Cramp (Cramp, 1985)]. Hunting can be targeted entirely acoustically, enabling effective location of prey even when hidden under leaves and in complete darkness (Payne, 1971; Hausmann et al., 2008). What role might the long legs play in hunting? For instance, might the legs be used to simply dissipate energy during the strike (Payne, 1971) with minimal loading to the owl – allowing high-speed dives and rapid contact with the prey while minimizing damage to the owl – or is the strike deliberately forceful? In order to study some functions of the legs in hunting, we derived ‘leap’ and ‘strike’ kinetics from forceplate measurements of a 10-year-old 0.219 kg barn owl ‘Kensa’ trained to target a stiff plastic beeper box (75×50×27 mm, length × width × depth). The box was hidden inside a ring of tall grasses (Fig. 1B) and mounted directly to the top plate of a Kistler 9287B forceplate (900×600 mm). A further six similar forceplates arranged in line allowed measurement of a subset of leaping take-offs. The primary forceplate and surrounding area was covered in short grass turf (except between box and top plate), as experience had shown that the owl has difficulty locating the beeper box in certain indoor environments, presumably for acoustic reasons. The owl was motivated to leap, circle briefly and pounce on – ‘strike’ – the beeper box (Fig. 1B) through prior training and food reward.

## RESULTS AND DISCUSSION

Seven take-off leaps and 19 strike trials were recorded and analysed (Fig. 1B–K), along with a single opportunistic recording of an ‘alighting’ landing when the owl elected not to strike. Leaping performance was impressive, but unexceptional, with peak forces of 5.7 body weights (Fig. 1E), peak instantaneous power of ~153 W body kg^−1^ (Fig. 1G) and work during the push-off up to 10 J body kg^−1^ (Fig. 1H, Table 1), values largely indistinguishable from those reported using similar techniques for a jumping guinea fowl *Numida meleagris* (Henry et al., 2005) of (means) 5.3 body weights, 145 W body kg^−1^ and 8.4 J kg^−1^, respectively. In both owl and guinea fowl, the leaps produced sufficient energy for up to 1 m vertical jumps.

What is more noteworthy is that this leaping performance was observed in a much smaller bird: the owl is 1/6.5 the mass of the reported 1.24 kg guinea fowl. Bennet-Clark (Bennet-Clark, 1977) describes why small animals, if they have absolutely short legs, have brief push-off times, and are thus muscle-power-limited and unable to leap as high unless elastic recoil can be used. The owl achieves leaping performance similar to that of the guinea fowl while undergoing similar deflections in the centre of mass (CoM) during the push-off phase (~0.2 m; Fig. 1K). Thus, the ‘functional’ leg extension of the owl (i.e. the deflection of the CoM during push off, and so the period during which power can be applied) is similar to that of the guinea fowl, despite its considerably smaller mass. This may be partly attributed to the relatively long legs of the owl (9% longer than a guinea fowl predicted from geometric similarity; see Materials and methods), the potential for input of work through wing elevation [not considered to be the case in the guinea fowl (Henry et al., 2005)] and the pitch-up motion of the body from a crouched starting position. Were the leaps to be powered entirely by leg muscles – which in fresh specimens account for 13.8% of body mass (Hartman, 1961) (11.2% in a road-kill bird) – then the instantaneous power requirements would exceed 1000 W muscle kg^−1^, and some mechanism of ‘power amplification’ might be indicated. However, some motion other than the extension of the legs is required to account for the CoM deflection during the leap push-off, as the sum of the femur, tibia and metatarsus is smaller (0.186 m for a 0.306 kg road-kill barn owl) than the CoM deflection. Thus, musculature associated with pitching and wing elevation presumably helps to power the leap. While the supracoracoideus (usually considered a major driver of wing elevation) is notably small in barn owls [highlighted by Hartman (Hartman, 1961), where 0.62% body mass is reported (0.56% in the road kill individual)], other wing muscles (Hartman reports 9.16% of body mass) may play a relevant role in driving the wings (18.1% body mass in road kill owl) – and so the CoM – upwards. Given the potential for non-leg muscles to contribute to

powering the leap, the question of whether there is relevant contribution of elastic recoil ‘power amplification’ mechanisms, or whether direct muscle action in a countermovement jump (owls were seen to dip before take-off, Fig. 1B, consistent with the force traces, Fig. 1E) is sufficient, cannot be answered from this study.

Starlings, finches and doves taking off from perches have been observed to use considerably lower proportional leg impulses, forces and works (for review, see Bonser, 1999) (see also Earls, 2000; Provini et al., 2012). So, why do owls choose to use such an energetic leap to become airborne? Possibly because they can – the ground is a reliable, solid surface to push against [quail reach up to 7.8 body weights (Earls, 2000)]. Further, owls have relatively large leg muscles [both barn owl and quails close to 14% body mass; starlings and doves up to ~8% (Hartman, 1961)]. But also the energetic leap may complement the acoustic hunting strategy of barn owls: with a powerful push-off, flaps, at least initially, need be less vigorous, thus making smaller (and quieter) disturbances of the air and nearby grass.

Strikes involved similar or greater impulses (Fig. 1C) and changes in energy (Fig. 1J), but during briefer periods and over shorter CoM deflections (Fig. 1K), requiring higher forces (Fig. 1D) and power magnitudes than leaping [maximum mean power during periods defined as actively pushing (see Materials and methods) were over triple those observed during leaping; see Table 1]. Force traces and some of their immediate derivatives cannot be viewed as perfect measures of the demands on the bird's muscles – contact with the surrounding grass prior to foot contact can be identified in force traces (Fig. 1F), and uncritical analysis of instantaneous force and power containing high-frequency vibrations (Fig. 1F,H) could be most misleading. We choose to present the force and power traces without application of a digital filter; the only signal conditioning is a single pole resistor–capacitor filter applying a −3 dB lowpass cut-off at 100 Hz prior to analogue–digital conversion. Peak force and power measurements are thus a little subjective (most explicit, objective measures of peaks would be highly sensitive to the filtering cut-offs or similar assumptions). However, inspection of the force and power

**Table 1.**
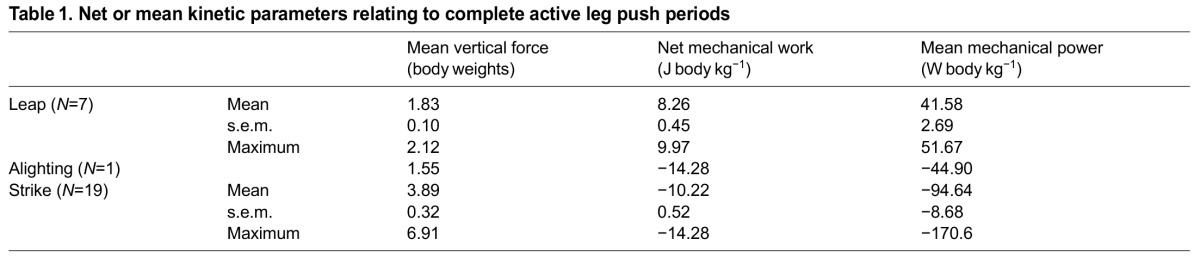
Net or mean kinetic parameters relating to complete active leg push periods

waveforms indicate peak forces during the strike – in terms that appear relevant to the legs – of 14.5 body weights and dissipative powers of ~500 W body kg^−1^. These considerably exceed peak values observed during leaping take-off.

These higher strike versus leap forces of the owl contrast with those of birds (and also primates) studied landing on perches, in which peak forces are considerably lower than during take-off (Bonser, 1999). Might the fact that the owl is landing on the ground – a substrate that is less likely to fail than a branch or twig – account for the high landing forces during the strike? Two observations point to this not being the case, and the strike being deliberately forceful: first, higher CoM deflections were observed during leaping take-off, and second (albeit with only a single observation), CoM deflections were also higher during a non-strike alighting landing (Fig. 1K). Thus CoM deflections during the strike could be greater, spreading the time for the impulse and reducing the forces and powers.

Why might the owl choose to apply high forces during the strike? A 0.2 kg owl pouncing with a force of 15 body weights would load a 20 g mouse with 150 mouse body weights – equivalent to an 80 kg man being squashed by the entire weight of a 12 tonne truck. However, while such forces may potentially be damaging to the prey, scaling issues mean this cannot be assumed; indeed, Payne (Payne, 1971) reports that the ‘strike’ was not sufficient to kill or even stun a mouse adequately to stop it from moving (though this observation was made with a slightly different setup). The process of subduing and killing the prey does not appear challenging to an owl, equipped with talons, an incapacitating squeeze (Payne, 1971), a hooked beak, and in any case capable of swallowing the prey whole. The ability to quickly penetrate leaf litter or snow may be a more realistic benefit of the high strike forces we observed; greater than 7 cm snow coverage is cited as a factor limiting owl distribution (Cramp, 1985).

## MATERIALS AND METHODS

Lengths for the bones dominating leg length of a 0.306 kg road-kill barn owl were determined from 3D models (Mimics, Materialise, Leuven, Belgium) based on computed tomographic data sets (GE Lightspeed, GE Medical, Milwaukee, WI, USA) and compared with those from a 1.21 kg guinea fowl (Kambic et al., 2014). For the owl and guinea fowl, respectively, the lengths were 50.7 and 79 mm for the femur; 81.8 and 116 mm for the tibia; and 53.9 and 73 mm for the metatarsus. The distance between the last thoracic vertebra and the centre of the hip joint gives some measure of back lengths for owl and guinea fowl: 81.7 and 94 mm, respectively. Owl muscle and wings were weighed after dissection. While in broad agreement with the measurements of Hartman (Hartman, 1961), we view the measurements from that study as more dependable (Hartman's study was based on eight fresh specimens).

Forces were sampled at 1000 Hz from a linear array of seven Kistler 9287B forceplates. The requirements of achieving good, repeatable performance from the owl meant that limb forces may have been somewhat attenuated because of the short turf covering of the plates near the beeper box. Further, some non-leg forces (between the wing or body and longer grass stems) may also be transmitted. In addition, vibrations make calculation of peak force and powers (especially during the strike) somewhat subjective. However, key net or time-averaged metrics (net work and impulse; average force and power) for ‘active’ push durations are relatively insensitive to these issues. We believe that any systematic measurement error (due, for instance, to force attenuation) will result in either little error or conservative, under-calculation of these parameters.

Forces (divided by body mass) provided CoM accelerations that were integrated forward (leap) or backward (strike and alight) with respect to time to provide velocities and CoM motions assuming zero velocity at either body weight before counter-movement (take-off) or steady body weight after landing. Integration periods were brief (~0.1 s), so integration drift was not considered an important source of error.

The ‘active’ push-off period was defined for take-off as the period between the force minimum due to the counter-movement, and the force minimum at the end of take-off. Take-off was complete before the wings initiated downstroke (see supplementary material Movies 1, 2); further, forces due to aerodynamic downwash can be distinguished in the force records (Fig. 1E), and appeared discretely after the direct-contact take-off period. Active push periods were defined subtly differently for strike and landing in an attempt to remove the inclusion of owl–grass contact and provide a robust objective measure despite vibrations after landing (the reverse not being present during take-off). Active push initiation in landing was defined as the first instant of vertical force falling below body weight on a time-reversed trace, starting at the peak force; termination was defined as the first instant vertical force fell below body weight after peak force.

## Supplementary Material

Supplementary Material
