# Supplementary material for: Leap and strike kinetics of an acoustically ‘hunting’ barn owl (Tyto alba)
Source: J Exp Biol. 2014 Sep 1;217(17):3002–5. doi: 10.1242/jeb.107169 (PMC4148188; doi:10.1242/jeb.107169)
Supplement: Supplementary Material [file supp_217_17_3002__index.html]

Leap and strike kinetics of an acoustically ‘hunting’ barn owl (Tyto alba) — Supplementary Material 

# Leap and strike kinetics of an acoustically ‘hunting’ barn owl (*Tyto alba*)

## JEB107169 Supplementary Material

**Files in this Data Supplement:**

- **Supplementary Material**
