# Supplementary material for: Leap and strike kinetics of an acoustically ‘hunting’ barn owl (Tyto alba)
Source: J Exp Biol. 2014 Sep 1;217(17):3002–5. doi: 10.1242/jeb.107169 (PMC4148188; doi:10.1242/jeb.107169)
Supplement: Supplementary Material [file supp_217.17.3002_JEB107169.pdf]

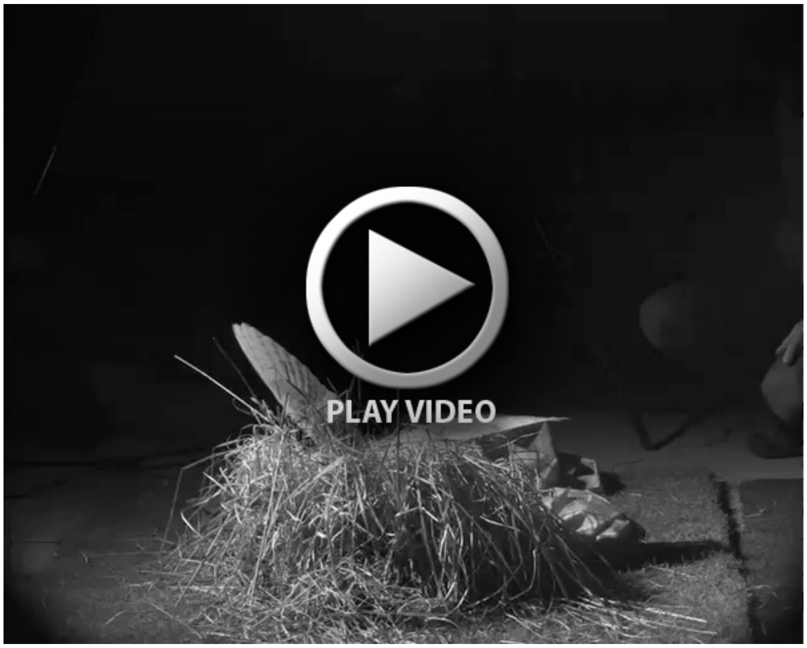

Movie 1. High speed video (no audio) of a complete leap/strike trial. Recorded at 500 frames s^{-1}; displayed at 24 frames s^{-1}.

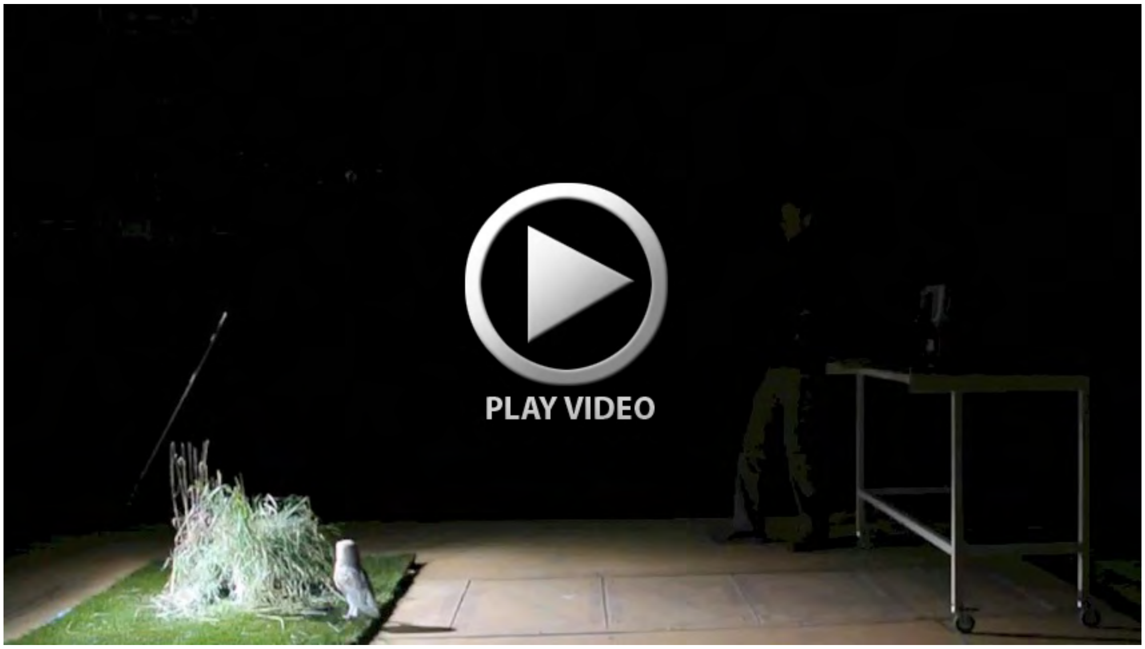

Movie 2. Video and audio of a leap/strike trial. Recorded at 30 frames s^{-1}; displayed (video and audio) at quarter speed.
